# Supplementary material for: Replication defective viral genomes exploit a cellular pro-survival mechanism to establish paramyxovirus persistence
Source: Nat Commun. 2017 Oct 6;8:799. doi: 10.1038/s41467-017-00909-6 (PMC5630589; doi:10.1038/s41467-017-00909-6)
Supplement: Supplementary file 1 — Supplementary Information [file 41467_2017_909_MOESM1_ESM.pdf]

Supplementary Figure 1

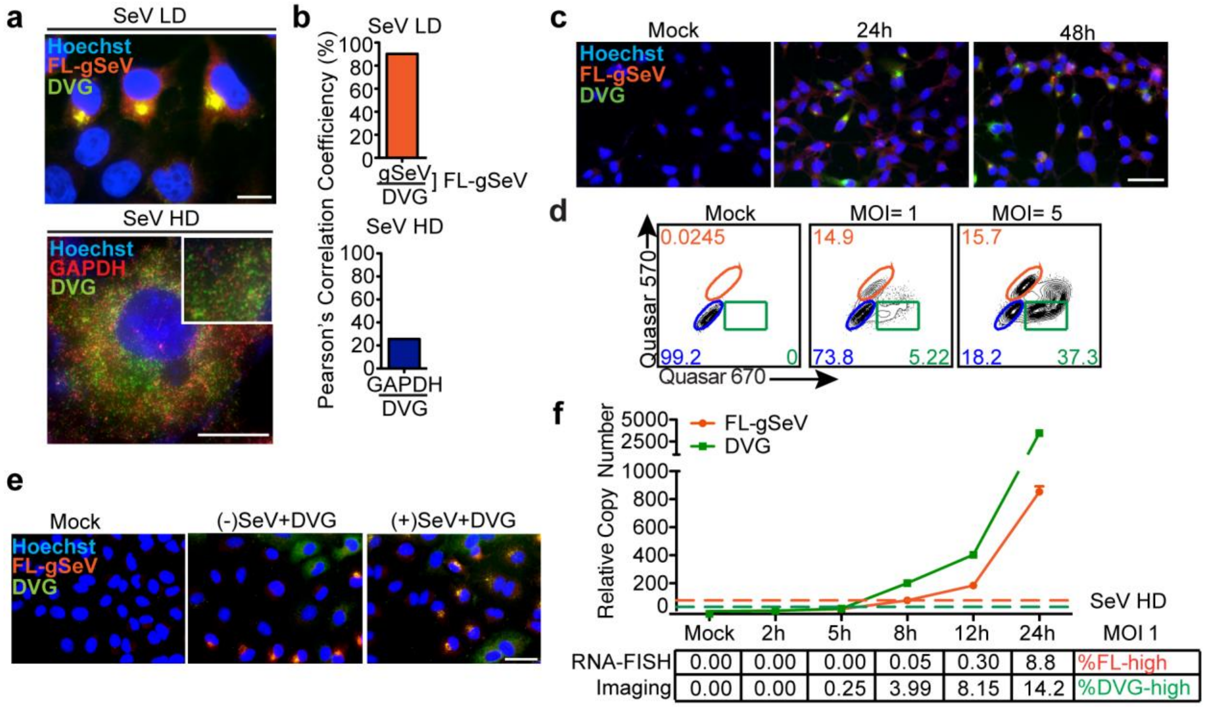

Supplementary Figure 1: Quality control of RNA FISH probes for DVG and gSeV detection. (a) Representative RNA FISH images of A549 cells infected with SeV LD (MOI=1.5 $TCID_{50}$/cell for 24 h, as positive control) or HD (MOI=5 $TCID_{50}$/cell for 6 h) after hybridization with probes designed against the 5' end of the viral genome (DVG, Quasar-670-labeled), and probes designed against the 3' end of the viral genomes (FL-gSeV, Quasar-570 labeled) or against host *GAPDH* mRNA. Magnification: SeV LD 100X. Bar: 10µm. The SeV HD infection panel was cropped from a 60X magnification image. (b) Co-localization efficiency of Quasar-570 labeled probes with gSeV or GAPDH mRNA with Quasar-670 labeled DVG probes in SeV LD and HD infected cells shown in (a). (c) Merged representative RNA FISH images of TC-1 cells infected with SeV HD (MOI=1.5 $TCID_{50}$/cell) for the indicated times. Magnification: 40X. Scale bar =20 µm. (d) A549 cells were infected with SeV HD at either a MOI of 1 or 5 $TCID_{50}$/cell for 24 h. Representative RNA FISH-FLOW graph. Gates correspond to non-detected (ND, blue), FL-gSeV-high cell (FL-high, orange), and DVG-high cells (DVG-high, green). Related to Fig.1e. (e) Merged representative RNA FISH image of A549 cells infected with SeV HD (MOI=1.5 $TCID_{50}$/cell) for 24 h. Magnification: 40X. Scale bar = 20µm. (f) A549 cells were infected with SeV HD (MOI=1 $TCID_{50}$/cell) and collected at the indicated time points. Copy numbers of (+)FL-gSeV and (+)DVG were quantified by RT-qPCR (line graph) while percentage of FL-high and DVG-high within the infected culture were quantified by RNA-FISH imaging (table). The experiment was independently repeated twice. RT-qPCR data are expressed as the copy number relative to the housekeeping gene *GAPDH* mRNA. See also the Supplementary Methods section.

Supplementary Figure 2

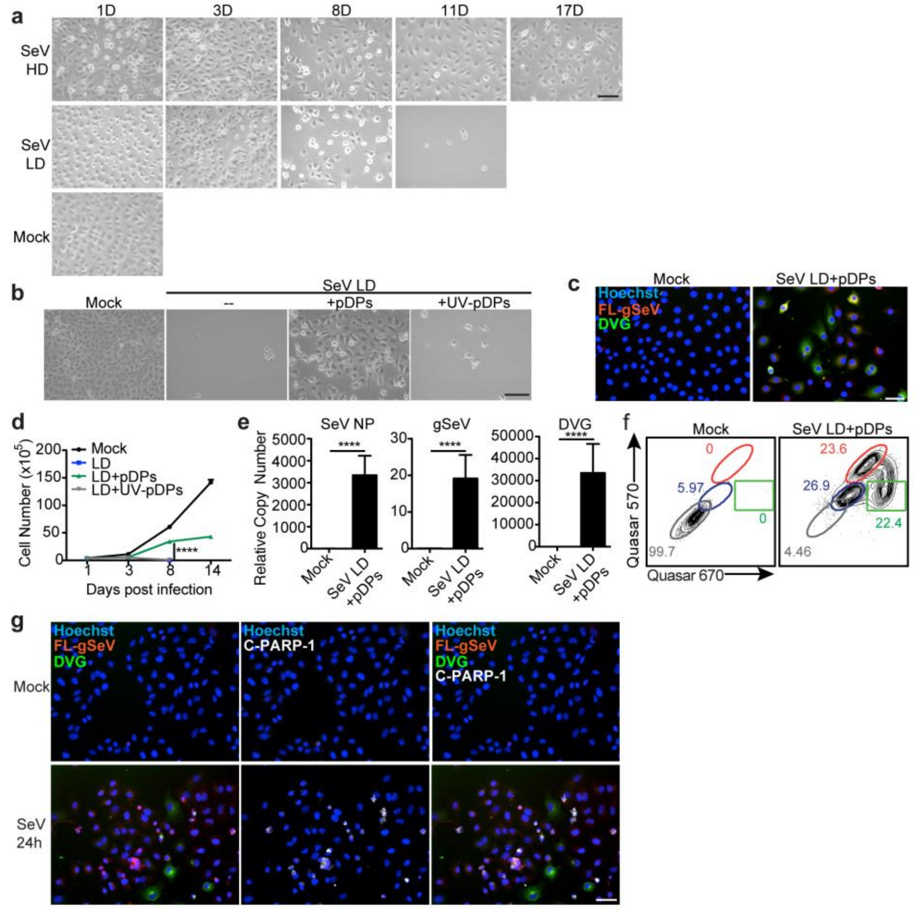

Supplementary Figure 2: DVG-enriched cells are less prone to SeV induced apoptosis compared to FL-gSeV-enriched cells. (a) Bright field images corresponding to RNA FISH images shown in Fig. 2a. One representative repeat is shown. Magnification: 20X. Scale bar = 20µm. (b-f) LLC-MK2 cells were mock infected or infected with SeV LD alone or in the presence of pDPs or UV inactivated pDPs (HAU = 300) and subcultured for 14 days. (b) Bright field images at 8 days post infection. One representative field from each treatment is shown. Magnification: 20X. Scale bar = 20µm. (c) Representative RNA FISH images of LLC-MK2 cell mock infected or infected with SeV LD+pDPs survived from death crisis at day 14 post infection. (d) Total cell numbers during the infection time course. (mean ± s.e.m.; n=3). (e) Expression of SeV NP mRNA, FL-gSeVs, and DVGs in survivor cells at day 14 post infection. (mean ± s.e.m.; n=3). (f) Representative RNA FISH-FLOW analysis of LLC-MK2 cells at day 14 after mock or SeV LD+pDPs infection. The percentages of ND (blue), FL-high (orange), and DVG-high (green) in the culture are indicated. (g) Representative RNA FISH-IF image for C-PARP-1 protein staining of A549 cells 24 h post SeV HD infection. Quantification of the images is shown in Fig. 2i. Hoechst signals were merged either with RNA FISH probes signals (left), C-PARP-1 signals (middle) and all channels (right). ****$p<0.0001$ by two-way ANOVA analysis with Bonferroni *post hoc* analysis (d) or unpaired t-test (e).Magnification: 20X. Scale bar =20µm. See also the Supplementary Methods section.

Supplementary Figure 3

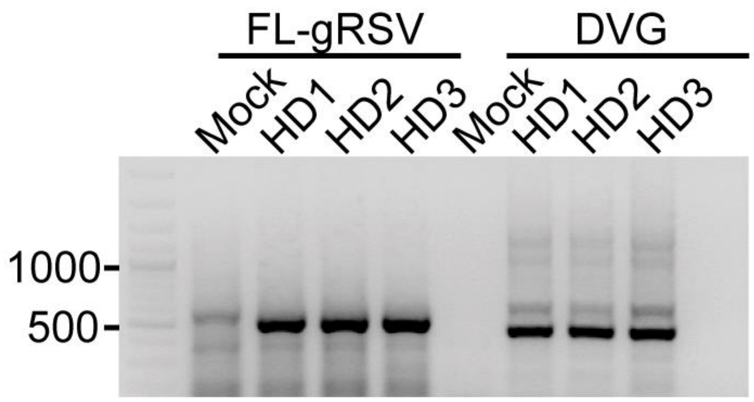

Supplementary Figure 3: DVG promote persistent RSV infection. PCR detection of FL-gRSV and DVGs in RSV HD infected A549 cells on day 23 post-infection. Samples from three different experiments (HD1, HD2 and HD3) and a non-infected control (Mock) are shown. See also the Supplementary Methods section.

Supplementary Figure 4

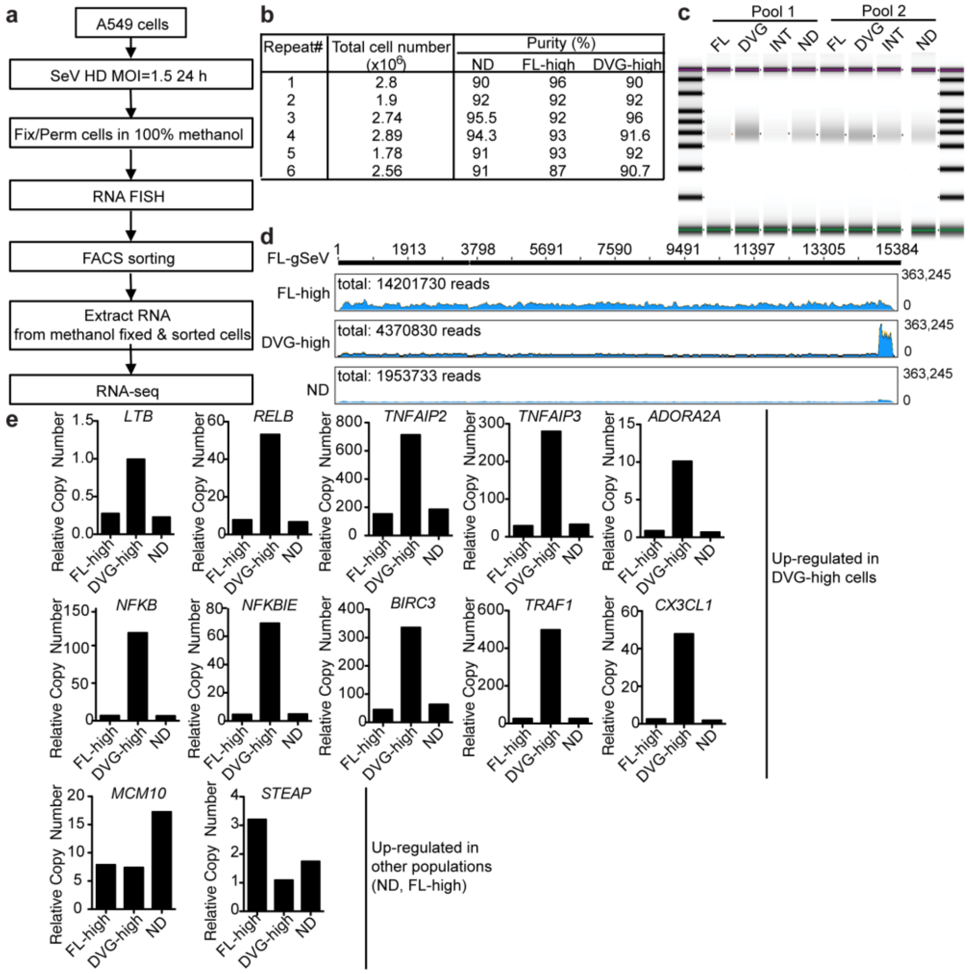

Supplementary Figure 4: Quality control of transcriptional profiling based on cells sorted by DVG and FL-gSeV content. (a) Flow chart of sample preparations for RNA-seq analysis. (b) Quality control of six independently repeated sorting prepared for RNA-seq. Total cell number after sorting and sorted cell purity of each sub-population are shown. (c) Quality control of cDNA samples by Qubit assay after reverse transcription. Pool 1 includes samples 1-3 and Pool 2, samples 4-6. (d) Integrated Genome Viewer representation of SeV Cantell strain (positive sense) reads from FL-high, DVG-high and ND cells sorted from SeV HD infected A549 cells. Histogram shows synopsis of total coverage in any given position of FL-gSeV (illustrated in black bar on top of the graph). Number on the right side of the graph represents the total reads at the position with highest coverage. Number of total reads aligning to SeV Cantell strain from each sub-population is indicated in the upper left corner of each histogram. (e) Gene expression verification by RT-qPCR of selected genes among differentially expressed genes after multiple comparison analysis as shown in Fig. 4a. Data are expressed as copy number relative to the housekeeping gene *GAPDH* averaged from two pools.

Supplementary Figure 5

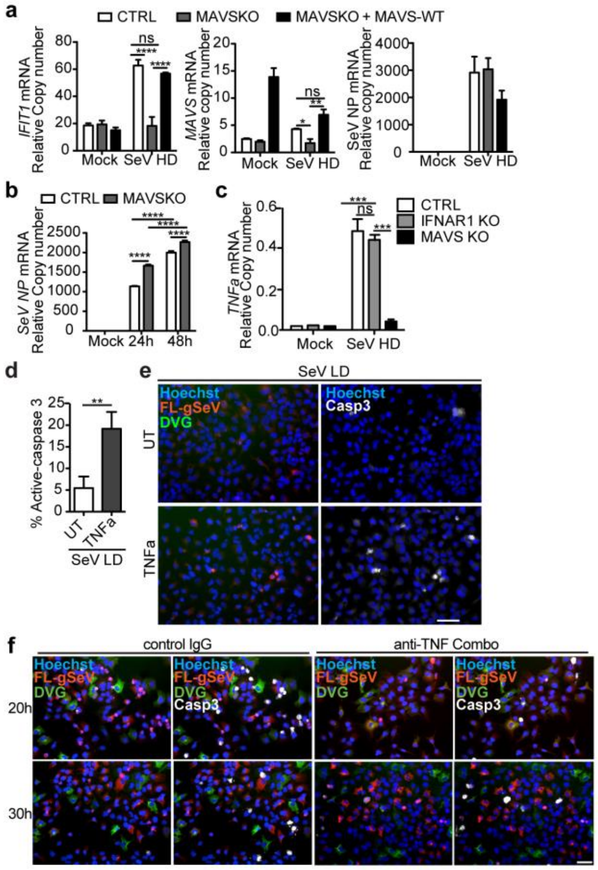

Supplementary Figure 5: DVGs dictate the function of TNFα during SeV infection. (a) CTRL and MAVS KO A549 cells were mock transfected or transfected with MAVS-WT expression plasmid for 6 h before infection with SeV HD (MOI=1.5 $TCID_{50}$/cell). Expression of IFIT1, MAVS, and SeV NP mRNA was quantified by RT-qPCR. (b) Expression of SeV NP mRNA in CTRL and MAVS KO A549 cells at 24 and 48 h post SeV HD infection. Data are expressed as the copy number relative to the housekeeping gene *GAPDH*. (c) TNFα mRNA expression from A549 CTRL, IFNAR1 KO, and MAVS KO cells mock infected or infected with SeV HD (MOI=1.5 $TCID_{50}$/cell) for 24 h. Data are expressed copy number relative to the housekeeping gene *GAPDH*. (d,e) A549 MAVS KO cells were infected with SeV LD (MOI=1.5 $TCID_{50}$/cell) for 16 h and then left untreated (UT) or treated with TNFα (10 ng/ml) for 8 h. (d) Percentage of apoptotic cells in each condition. (e) Representative RNA FISH-IF images of active-caspase 3 protein (white), FL-gSeV genome (orange) and DVGs (green) staining of infected cells. Hoechst signals were merge respectively with RNA FISH probes signals (left columns) and active-caspase 3 (right columns). (f) RNA-FISH-IF staining of A549 cells infected with SeV HD (MOI=1 $TCID_{50}$/cell) and incubated with control IgG antibody or antibodies against TNFα, TNFR1, or and TNFR2 (anti-TNF Combo). Hoechst signals were merged with RNA FISH signals (left columns) or all channels (right columns). Magnification: 20X. Scale bar =20μm. Values represent mean ± s.e.m. from 3 independently repeated experiments, unless indicated. ***$p<0.001$, ****$p<0.0001$ by two-way ANOVA with Bonferroni *post hoc* analysis (a-c). **$p<0.01$ by student's t test (d). ns=non-significant.

Supplementary Figure 6

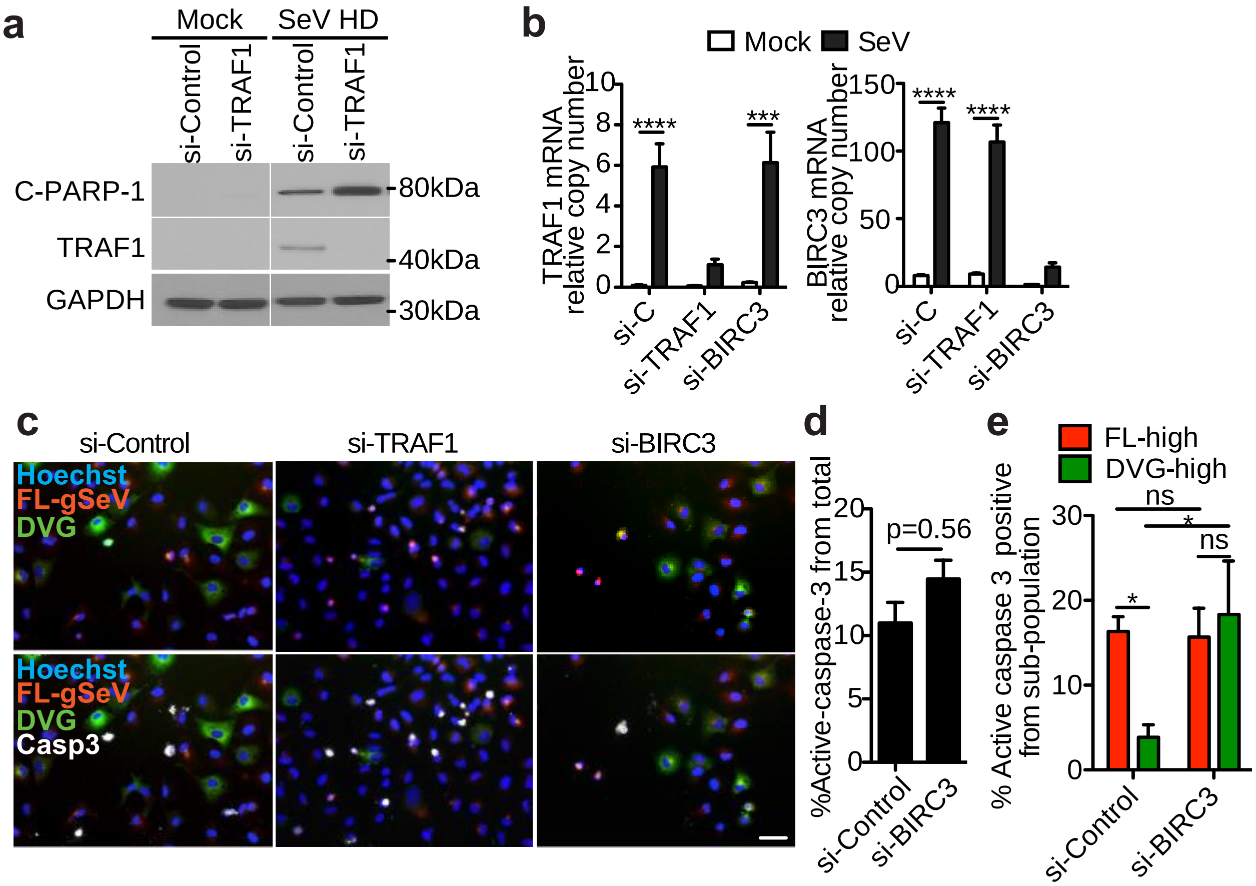

Supplementary Figure 6: TRAF1 and BIRC3 knockdown enhance apoptosis of DVG-high cells. A549 cells were transfected with either control siRNA (si-Control) or *TRAF1* siRNA (si-*TRAF1*) and infected with SeV HD. (a) Western Blot analysis of si-Control and si-TRAF1 treated cells infected with SeV HD to confirm specific knockdown of TRAF1 protein. (b) Expression of TRAF1 and BIRC3 mRNA in si-Control, si-TRAF1, or si-BIRC3-treated cells infected with SeV HD to confirm specific transient silencing of TRAF1 and BIRC3 expression, respectively. Gene expression is shown as copy number relative to a house keeping gene GAPDH expression. Error bars indicate mean ± s.e.m. of three independent experiments ***$p<0.001$, ****$p<0.0001$ by two-way ANOVA with Bonferroni post hoc test. (c) Representative RNA FISH-IF images of active-caspase 3 staining 24 h after SeV HD infection (MOI=1.5 $TCID_{50}$/cell) of cells treated with si-Control, si-TRAF1, or si-BIRC3. Hoechst signals were merged with RNA FISH probes signals (upper panel) or all channels (lower panel). Magnification: 20X. Scale bar = 20µm. (d) Percentage of apoptotic cells within the total cell cultures treated with si-Control or si-BIRC3 (mean ± s.e.m.; n=3, unpaired t-test). (e) Percentage of active-caspase 3 positive cells within FL-high and DVG-high cells in the same conditions shown in d. (mean ± s.e.m.; n=3). *$p<0.05$ by two-way ANOVA with Bonferroni's *post hoc* test. ns=non-significant. See also the Supplementary Methods section.

Supplementary Table 1 Primers used in RT-qPCR assays.

Name	Forward (5'-3')	Reverse (5'-3')
TRAF1	ctgtgcaggctgtctctctg	cggcttcctgggcttatag
LTB	ggcggtgcctatcactgt	gaaaccccagtccttgctg
TNFAIP3	tgcacactgtgtttcatcgag	acgctgtgggactgactttc
NFKBIE	ctcatccactctgtgcaagg	tcatcaaagggcaaaaggac
NFKB2	cacatgggtggaggctct	actggtaggggctgtaggc
RELB	aagaaaaagccggccatc	cacggtgccagagaagaagt
MCM10	caaggaaaagatggccagag	tccatatgctgaaggtttttcc
TNFAIP2	catcgccactgtagacacga	accaggtgcaggtgcaag
ADORA2A	ctacattgccatccgcatc	aacctagcatgggagtcagg
CX3CL1	ggctccgatatctctgtcgt	atgttgcatttcgtcacacc
BIRC3	gcttgaaaagactgggcttg	aagaagtcgttttcctcctttgt
TRAF2	ggaacacacctgtccctctt	ggtcgagcagcattaaggtc
STEAP3	gcctcagaccctcacgtc	gctactatcgctgtccaccag
GAPDH	gcaaattccatggcaccgt	tcgccccacttgattttgg
IFNB1	gtcagagtcgaaatcctaag	acagcatctgctggttgaag
SeV NP	tgccctggaagatgagttag	gcctgttggtttgtggtaag
SeV DVG	cctcaggttcctgatctcac	accagacaagagtttaagagatatgtatt
FL-gSeV	aatctaggtatctcactccatg	aagagattctcgagtatcagaa

Supplementary Table 2 Target sequence positions for (+)SeV RNA FISH probes.

Probe #	Position on (+)FL-gSeV genome (GenBank ID: AB855654.1)
gSeV 1	45-65
gSeV 2	99-18
gSeV 3	127-146
gSeV 4	466-485
gSeV 5	1363-1382
gSeV 6	2075-2094
gSeV 7	2150-2169
gSeV 8	2815-2834
gSeV 9	3646-3665
gSeV 10	4712-4731
gSeV 11	4827-4846
gSeV 12	5210-5229
gSeV 13	5314-5333
gSeV 14	5702-5721
gSeV 15	6389-6408
gSeV 16	6580-6599
gSeV 17	6901-6920
gSeV 18	7065-7084
gSeV 19	7270-7289
gSeV 20	7411-7430
gSeV 21	7444-7463
gSeV 22	7728-7747
gSeV 23	8421-8440
gSeV 24	9183-9202
gSeV 25	9938-9957
gSeV 26	10205-10224
gSeV 27	10746-10765
gSeV 28	11132-11151
DVG1	14944-14963
DVG2	14966-14985
DVG3	14990-15009
DVG4	15019-15038
DVG5	15041-15060
DVG6	15065-15084
DVG7	15109-15128
DVG8	15132-15151
DVG9	15169-15188
DVG10	15191-15210
DVG11	15213-15232
DVG12	15235-15254
DVG13	15258-15277
DVG14	15285-15304
DVG15	15321-15340

Supplementary Table 3 Target sequence positions for (-)SeV RNA FISH probes.

Probe #	Position on (-)FL-gSeV genome (Complementary sequence of GenBank ID: AB855654.1)
negSeV1	1960-1979
negSeV3	2185-2204
negSeV4	2594-2613
negSeV5	2736-2755
negSeV6	2938-2957
negSeV8	4396-4415
negSeV9	5024-5043
negSeV10	5459-5478
negSeV11	5634-5653
negSeV12	6134-6153
negSeV13	6610-6629
negSeV14	6892-6911
negSeV15	7021-7040
negSeV16	7088-7107
negSeV17	7239-7258
negSeV18	9252-9271
negSeV19	9830-9849
negSeV20	10183-10202
negSeV21	10305-10324
negSeV23	10805-10824
negSeV24	11184-11203
negSeV25	11624-11643
negSeV26	11780-11799
negSeV27	12220-12239
negSeV28	12998-13017
negSeV29	13666-13685

Supplementary Table 4 Target sequence positions for RSV RNA FISH probes.

Probe #	Position on (+)FL-gRSV genome (GenBank ID: AF035006)
gRSV 1	361-380
gRSV 3	718-737
gRSV 4	957-976
gRSV 6	1322-1341
gRSV 8	1738-1756
gRSV 9	2052-2071
gRSV 10	2267-2286
gRSV 12	2429-2448
gRSV 13	2912-2932
gRSV 15	3437-3456
gRSV 16	3872-3891
gRSV 17	4951-4970
gRSV 19	5657-5676
gRSV 20	6277-6296
gRSV 21	6713-6730
gRSV 22	6865-6884
gRSV 23	7036-7055
gRSV 24	7604-7623
gRSV 25	8103-8122
gRSV 26	8336-8335
gRSV 27	8917-8935
gRSV 28	10835-10854
gRSV 29	10972-10991
gRSV 30	11504-11523
gRSV 31	11637-11656
gRSV 32	11940-11959
DVG 1	14923-14942
DVG 3	15019-15038
DVG 4	15089-15108
DVG 5	15111-15130
DVG 6	15134-15153
DVG 7	15156-15175
DVG 8	15181-15200
DVG 9	15203-15222

Supplementary methods

RNA FISH probe specificity analysis.

For testing DVG RNA FISH probe specificity, A549 cells infected with SeV LD or SeV HD were fixed, permeabilized, and hybridized with Quasar-670 labeled DVG probes and Quasar-570 labeled gSeV probes or Quasar-570 labeled GAPDH probe at 125 nM concentration diluted in hybridization buffer. Hybridization was performed overnight in a humidified chamber at 37°C. Nuclear staining using 0.5 μg/ml of Hoechst 33342 was performed afterwards and the coverslides were mounted in GLOX anti-fade media. Imaging acquisition was performed with a Nikon E600 epifluorescence microscope equipped with a 100X, 1.4 numerical aperture oil immersion objective (Zeiss) and a Zeiss AxioCam MRm camera. Imaging acquisition of SeV HD samples was performed with a Nikon Ti-E inverted fluorescence microscope equipped with a 100X Plan-Apo objective (numerical aperture of 1.43) and with a cooled CCD camera (Andor iKon 934). Colocalization quantification was performed in Volocity Quantitation module (Perkin Elmer). Global Pearson Correlation (PCC) analysis was performed by drawing multiple regions of interest (ROI) corresponding to different cells and calculating an average. Quantification results of the representative images are shown in Supplementary Fig. 1a.

FISH probe sensitivity assay

A549 cells were infected with SeV HD at a MOI=1 $TCID_{50}$/cell and harvested 2, 5, 8, 12, and 24 h later for analysis. Fixed cells were then processed for RNA FISH as described in detail in the methods section of the main text using probes against FL-gSeV or DVGs. RNA FISH images were quantified and the percentage of FL-high or DVG-high cells was calculated and plotted in

function of time. (+)FL-gSeV and (+)DVGs were also quantified by RT-qPCR. For this, 1-2 μg of isolated total RNA were reverse transcribed using Superscript III without RNaseH activity to avoid self-priming (primer: 5'-GGTGAGGAATCTATACGTTATAC-3'). Recombinant RNase H (Invitrogen) was later added to the reverse transcribed samples. cDNA was amplified with viral product-specific qPCR primers in the presence of SYBR green (Applied Biosystem) using primer set: (+)FL-gSeV (for: 5'-GACCAGGAAATAAAGAGTGCA-3'; rev: CGATGTATTGGCATATAGCGT-3') ; (+) DVG: (for: 5'-TCCAAGACTATCTTTATCTATGTCC-3'; rev: GGTGAGGAATCTATACGTTATAC-3'). Copy numbers were normalized to the housekeeping gene *GAPDH*. Detection threshold for both (+)FL-gSeV and (+)DVG were below 100 copies..

pDPs supplementation and long-term subculture.

pDPs were prepared as described in the method section of the main text and inactivated under UV light (254nn at 6 inches distance) for 6 min (UV-pDPs). For SeV infection with pDPs supplementation, LLC-MK2 cells were infected with SeV LD (MOI=1 $TCID_{50}$/cell) plus either pDPs or UV-pDPs (HAU=300). Infected cells were sub-cultured on days 3 and 8 post infection and harvested on day 14 post infection for endpoint analysis, methods for each assay are described in the method section of the main text.

Protein detection

The amount of TNFα in SeV infected A549 cells was measured by Enzyme-linked immunosorbent assay (ELISA) using a human TNFα detection kit (R&D system) according to the manufacturer's instructions. For western blots, whole cellular extracts were prepared from

pelleted cells in a NP-40-based lysis buffer containing phosphatase inhibitors, proteinase inhibitors (Roche and Thermo Scientific). Concentration of protein was measured by BCA assay (Themo Scientific). For each sample, 20 μg of protein extract were boiled for 5 min and resolved on 10% Bis-Tris precast gels (Bio-Rad) followed by transfer to a polyvinylidene fluoride (PVDF) membrane (Millipore). The membrane was blocked with 5% non-fat milk and immunoblotted with mouse anti-TRAF1 (1: 1000 dilution, Santa Cruz, sc-6253), mouse anti-GAPDH (1: 5000 dilution, Sigma, G-8795), or anti- mouse IgG (HRP-conjugated, 1:10000 dilution, Jackson Immunologicals, Cat#115-035-044). Lumi-Light western blotting substrate (Roche) was used for HRP detection according to the manufacturer's protocol.
